# Epiregulin reprograms cancer-associated fibroblasts and facilitates oral squamous cell carcinoma invasion via JAK2-STAT3 pathway

**DOI:** 10.1186/s13046-019-1277-x

**Published:** 2019-06-24

**Authors:** Yujia Wang, Yue Jing, Liang Ding, Xiaoxin Zhang, Yuxian Song, Sheng Chen, Xingxing Zhao, Xiaofeng Huang, Yumei Pu, Zhiyong Wang, Yanhong Ni, Qingang Hu

**Affiliations:** 1 Department of Oral & Maxillofacial Surgery Nanjing Stomatological Hospital, Medical School of Nanjing University, 30 Zhongyang Road, Nanjing, 210008 China; 2 Central Laboratory Nanjing Stomatological Hospital, Medical School of Nanjing University, 30 Zhongyang Road, Nanjing, 210008 China; 3 Department of Oral Pathology Nanjing Stomatological Hospital, Medical School of Nanjing University, Nanjing, China; 4 Department of Oral & Maxillofacial Surgery Sun Yat-Sen Memorial Hospital, Sun Yat-Sen University, Guangzhou, China

**Keywords:** Oral squamous cell carcinoma, Cancer-associated fibroblasts, Epiregulin, Transition, Metastasis, Invasion

## Abstract

**Background:**

Local resident normal fibroblasts (NFs) are the major source of cancer-associated fibroblasts (CAFs), which are distinguishable from NFs by their tumor-supportive properties. However, the mechanism and the effects underlying the transition of NFs to CAFs in oral squamous cell carcinoma (OSCC) remain unclear.

**Methods:**

Five pairs of matching primary NFs and CAFs derived from OSCC patients were sent for RNA sequencing. Epiregulin (EREG) expression was analyzed by IHC in fibroblasts from OSCC patients. The role of EREG in the NF-CAF transition and the consequential effects on OSCC progression were examined by upregulation/downregulation of EREG in NFs/CAFs both in vitro and in vivo.

**Results:**

Here, we identified epiregulin (EREG) as the most remarkably upregulated gene in CAFs. High EREG expression in CAFs correlated with higher T stage, deeper invasion and inferior worst pattern of invasion (WPOI) in OSCC patients and predicted shorter overall survival. Overexpression of EREG in NFs activated the CAF phenotype. Mechanistically, the JAK2/STAT3 pathway was enhanced by EREG in parallel with increased IL-6 expression, which could be inhibited by the JAK2 inhibitor AG490. Recombinant IL-6 upregulated the JAK2/STAT3/EREG pathway in a feedback loop. Moreover, EREG-induced CAF activation promoted the epithelial-mesenchymal transition (EMT) necessary for migration and invasion, which was dependent on JAK2/STAT3 signaling and IL-6. In vivo, EREG expression in stroma fibroblasts promoted tumor growth with high stromal α-SMA, phospho-JAK2/STAT3, and IL-6 expression and upregulated EMT in HSC3 cells.

**Conclusions:**

EREG is essential for the NF-CAF transformation needed to induce EMT of tumor cells in a JAK2-STAT3- and IL-6-dependent manner in OSCC.

**Electronic supplementary material:**

The online version of this article (10.1186/s13046-019-1277-x) contains supplementary material, which is available to authorized users.

## Background

It is widely accepted that tumor development depends on a complex interaction between malignant cells and their microenvironment [1]. Cancer-associated fibroblasts (CAFs), as a major cellular component of the tumor stroma, coevolve with cancer cells and contribute to cancer cell growth, survival, and metastasis [2, 3]. Often marked by smooth muscle actin (SMA), vimentin and N-cadherin, CAFs acquire a phenotype similar to that of myofibroblasts, which are activated in wound healing and fibrosis, with a morphology and function that differ from those of normal fibroblasts (NFs) [4]. In oral squamous cell carcinoma (OSCC), CAFs have been recognized as a poor prognostic factor, promoting invasion, metastasis, and recurrence of OSCC [5–8]. There are 5 cellular origins of CAFs, among which NFs are a major source [9, 10]. Despite the overwhelming consensus on the tumor-supportive role of CAFs, the molecular mechanisms underlying the transition from NFs to CAFs remain largely unclear. Further clarification of the mechanism underlying the transition from NFs to CAFs is important for cancer diagnosis and therapy.

Epiregulin (EREG), which is mainly secreted by fibroblasts, is a member of the epidermal growth factor (EGF) family of peptide growth factors and promotes tumor development, migration and invasion [11–14]. It has been reported that EREG expression is increased in healing wounds [13, 15]. However, whether EREG is involved in the NF-CAF transition remains unknown. As CAFs function similarly to myofibroblasts in wound healing, we hypothesized that EREG might participate in the transition of NFs to the CAF phenotype from the very start of NF activation, thus promoting tumor progression.

This study aimed to determine the role of EREG in the NF-CAF transition in the progression of OSCC and to identify the underlying mechanism. By understanding the NF-CAF transition, we can improve our knowledge of the supportive role of the tumor stroma, thus providing new insights into cancer treatment.

## Material & Methods

### Primary NFs/CAFs separation, cultivation and RNA-sequence

OSCCs, and matching normal oral biopsies were collected following ethical approval (2017NL-013(KS)) and written informed consent. 11 matching CAFs, and NFs were isolated according to a method previously described [16] and forwarded for morphologic examination. After appropriate NFs/CAFs were isolated and chosen, 5 pairs of matched NFs and CAFs were sent for library construction, and RNA sequencing was performed.

We performed the RNA-seq with the help of Novel Bioinformatics Co., Ltd. (Shanghai, China). Firstly, total RNA was extracted by Trizol reagent (Invitrogen) separately. The RNA quality was checked by Bioanalyzer 2200 (Aligent) and kept at − 80 °C.The RNA with RIN > 8.0 is right for cDNA library construction. Secondly, the complementary DNA (cDNA) libraries for single-end sequencing were prepared using Ion Total RNA-Seq Kit v2.0 (Life Technologies). The cDNA libraries were then processed for the Proton Sequencing process according to the commercially available protocols. Thirdly, Mapping of single-end reads. The clean reads were then aligned to human genome (version: GRCh38.p1) using the MapSplice program (v2.2.0). Finally, pathway analysis was performed and we also applied EBseq algorithm to filter the differentially expressed genes, the significant analysis and FDR analysis were performed. Besides, we presented gene co-expression Networks to find the relations among different mRNA and LncRNA. Results of LncRNA have been reported in a previous publication [17]. Results of mRNAs with the most significant changes are listed in Additional file 1: Table S1. The data in the list are the mean value of the 5 pairs of samples.

### Patients and tissue samples

For the analysis of expression of EREG, 104 consecutive unselected T_1-4_N_0_M_0_ OSCC patients treated in the Department of Oral and Maxillofacial Surgery at Nanjing Stomatology Hospital from 1 January, 2009 to 31 December, 2010 were included into the study. Including criteria: (1) diagnosed with primary OSCC by hematoxylin and eosin staining, and staged as T_1-4_N_0_M_0_ according to the 8th version of American Joint Committee on Cancer (AJCC) TNM staging system [18] by experienced pathologists from the Department of Pathology at Nanjing Stomatology Hospital; (2) underwent primary surgical treatments in the Department of Oral and Maxillofacial Surgery at Nanjing Stomatology Hospital; (4) paraffin-fixed sample available; (3) with complete clinical-pathological information and follow-up data. Excluding criteria: (1) diagnosed with autoimmune or other malignant diseases and pregnant; (2) had adjuvant treatment before or after surgery. The ethical approval for this study was obtained from the Research Ethics Committee of Nanjing Stomatology Hospital (approval number: 2017NL-013(KS)). The clinical pathologic characteristics of the 104 OSCC patients are listed in Table 1.

**Table 1:** Clinical pathologic characteristics and chi-square analysis of OSCC patients

Clinical Variables	Total (%)	EREG in CAFs		χ^2^	*p* value	OR	95% CI
		Low (%)	High (%)				
Total	104 (100)	61 (100)	43 (100)	–	–	–	–
Sex							
Male	51 (49.0)	20 (32.8)	31 (72.1)	15.59	< 0.0001***	0.1888	0.08035 to 0.4438
Female	53 (51.0)	41 (67.2)	12 (27.9)				
Age							
<60	44 (42.3)	21 (34.4)	23 (53.5)	3.755	0.0527	0.4565	0.2053 to 1.015
≥60	60 (57.7)	40 (65.6)	20 (46.5)				
TNM stage							
T_1-2_N_0_M_0_	80 (76.9)	53 (86.9)	27 (62.8)	8.248	0.0041**	3.926	1.492 to 10.33
T_3-4_N_0_M_0_	24 (23.1)	8 (13.1)	16 (36.2)				
Differentiation							
Well	67 (64.4)	43 (70.5)	24 (55.8)	2.371	0.1236	1.891	0.8366 to 4.275
Medium to poor	37 (35.6)	18 (29.5)	19 (44.2)				
Perineural Infiltration							
No	88 (84.6)	55 (90.2)	33 (76.7)	3.489	0.0618	2.778	0.9241 to 8.350
Yes	16 (15.4)	6 (9.8)	10 (23.3)				
DOI							
<5 mm	62 (59.6)	48 (78.7)	14 (32.6)	22.29	< 0.0001***	7.648	3.157 to 18.53
≥5 mm	42 (40.4)	13 (21.3)	29 (67.4)				
WPOI							
1–3	59 (56.7)	46 (75.4)	13 (30.2)	20.97	< 0.0001***	7.077	2.954 to 16.96
4–5	45 (43.3)	15 (24.6)	30 (69.8)				

*DOI* depth of invasion, *WPOI* worst pattern of invasion

### Immunohistochemistry staining and evaluation

Immunofluorescence of cryosections or immunohistochemistry of paraffin-embedded tissue was done as previously described [19]. Primary antibodies were rabbit anti-human EREG (Abcam), phospho-JAK2 (Proteintech), phospho-STAT3 (CST), SMA (Abcam).

EREG expression in CAFs was identified on the basis of SMA staining. CAFs were defined as SMA positive, spindled-shaped cells in the stroma (data of SMA staining not shown). EREG staining was assessed only in fibroblasts in tumor stroma by the evaluation of staining intensity and the percentage of EREG positive fibroblasts according to criteria modified from Zhang J et al. (Fig. 2a). Briefly, EREG expression was scored by a combination of staining intensity (score: 0 = no staining, 1 = weak staining, 2 = medium staining, 3 = strong staining) and percentage of EREG positive cells (score 0 = 0%~ 5% positive cells, 1 = 6%~ 33% positive cells, 2 = 34%~ 66% positive cells and 3 = 67%~ 100% positive cells) by a method modified from Zhang J, et al. (Fig. 2a) [1]. EREG staining index was taken as the product of staining intensity and percentage. The staining index was further divided into low and high: score 0–4 (negative to medium) was defined as EREG low, score 6 and 9 (strong) was defined as high EREG expression. Two observers examined the images independently without the knowledge of patients’ information.

### Construction of plasmid and siRNA

EREG over expression plasmid was purchased from Genechem Co.,Ltd. (Shanhai, China). The plasmid served as vector was pcDNA3.1+ (Amp). Amplified DNA fragments were inserted into the EcoR I/Xhol sites of pcDNA3.1+ vector. The amplification sequences were: EREG-F: GAATTCatgaccgcggggaggaggatggag; EREG-R: CTCGAGtcagacttgcggcaactctggatc. Empty plasmid was used as control.

The siRNAs were synthesized by Ribobio Co. Ltd. (Guangzhou, China) with following sequences: siNC: 5′-UUCUCCGAACGUGUCACGUdTdT-3′, siEREG-1: 5′-CCAGGAGAGUCCAGUGAUAdTdT-3′, siEREG-2: 5′-CCACCAACCUUUAAGCAAAdTdT-3′, siEREG-3: 5′-UACACUUUGUUAUUGACACUUdTdT-3′.

### Transient transfection

Plasmid-mediated overexpression of EREG in NFs, and siRNA-mediated interference of EREG in CAFs, were carried out using Lipofectamine® 2000 Transfection Reagent (Thermo Fisher Scientific) according to manufacturer’s instructions. Transient transfection was applied in all in vitro experiments.

### Quantitative reverse transcription PCR

Cells were lysed with TRIzol® reagent (Life Technologies), total RNA was extracted following the manufacturer’s instructions, and cDNA synthesis was conducted using High-Capacity cDNA Archive Kit system (Applied Biosystems). Quantitative PCR (q-PCR) was then conducted using iTaq™ Universal SYBR® Green Supermix (BIO-RAD). Comparative 2^-△Ct^ or 2^-△△Ct^ method was used to quantify the relative mRNA expression as indicated.

### Western blotting

Western blots were performed using an SDS–PAGE electrophoresis system as described previously [19], employing rabbit anti-human EREG (Abcam), rabbit anti-human SMA (Abcam), rabbit anti-human E-cadherin (CST), rabbit anti-human N-cadherin (CST), rabbit anti-human vimentin (Proteintech), rabbit anti-human p-JAK2 (CST), rabbit anti-human JAK2 (CST), rabbit anti-human p-STAT3 (CST), mouse anti-human STAT3 (CST), rabbit anti-human p-NF-kB (CST), rabbit anti-human NF-kB (CST), rabbit anti-human p-p38 (CST), rabbit anti-human p38 (CST), rabbit anti-human p-akt (CST), rabbit anti-human akt (CST), rabbit anti-human p-erk1/2 (CST), rabbit anti-human erk1/2 (CST), rabbit anti-human snail (CST), antibodies. The blots were re-probed for rabbit anti-human GAPDH (Bioworld) to control for protein loading and transfer.

### Immunofluorescence

Immunofluorescence analyses were carried out as previously described [20]: primary antibodies, goat anti-human EREG (R&D), rabbit anti-human SMA (Abcam) and mouse anti-human CK (Abcam); secondary antibodies, AlexaFluor 647 donkey anti-goat IgG and AlexaFluor 488 goat anti-rabbit 594 goat anti-mouse IgG (Life Technologies).

### Conditioned medium (CM) collection

NFs and CAFs were seeded into 12-well plates at identical density. NFs were transfected with control plasmid (pcDNA3.1) or EREG-overexpression plasmid (pcDNA3.1 EREG), and CAFs were transfected with control siRNA (siNC) or EREG siRNA (siEREG). The culture medium were collected 48 h after transfection, centrifuged at 3000RPM for 10 min to remove suspended cells, and stored in − 80 °C until Use.

### Elisa

Cells were transfected with over-expression plasmid or siRNA for EREG, followed with or without treatment of AG490 or rIL6 as indicated. After 48 h, the supernatants were collected for the measurement of IL6 with an ELISA kit (Dakewei, China) according to the manufacturer’s instructions.

### CCK8 assay

Primary NFs and CAFs were transfected with pcDNA3.1-control, pcDNA3.1-EREG siNC or siEREG as indicated. The treated cells were either seeded in 96-well, or their culture medium was used to treat HSC3 cells seeded in 96-well. Cell viabilities were determined at 0, 24, 48, 72 and 96 h after transfection (in case of primary NFs/CAFs) or CM treatment (in case of HSC3 cells).

### Colony formation assay

30/ml HSC3 cells were seeded in 12 well plates. Conditioned-medium from NFs + pcDNA3.1/pcDNA3.1-EREG, or CAFs+siNC/siEREG 24 h after transfection were used to treat HSC3 cells. The CM was changed every 3 days. After 1 to 2 weeks of cultivation, when visible colonies were formed, the medium was removed. Cells were 5% formalin-fixed after washed with PBS for 3 times, then stained with crystal violet.

### Wound healing assays

HSC3 cells were seeded in 12-well plates until 100% fusion. After overnight starvation with serum-free DF12 medium, a scratch was made using a micropipette tip and cells were washed to remove detached cells and debris. Photographs of the same area of the wound were taken at 0 and 18 h for measuring the closure of the wound after the treatment of the indicated serum-free CM from NFs/CAFs.

### Invasion and migration assays

The invasion and migration ability of HSC3 cells were tested in Transwell (boyden chamber) with an 8-um pore size of the polycarbonate membranes. Primary NFs and CAFs were placed in the lower chamber at a concentration of 3 × 10^4 cells/ml (0.6 ml/well). The NFs were then transfected with or without EREG-overexpression plasmids, and CAFs transfected with or without siEREG RNA. AG490 and recombinant human IL6 (Abcam) were added as indicated. After 5 h of incubation, the transfection mix was replaced by 0.5% FBS. Simultaneously, HSC3 cells, which had been previously starved overnight, were re-suspended with serum-free DF12 culture medium at a concentration of 10^6 cells/ml and then added to the upper chamber (200ul/well). For invasion assay, 40ul matrigel mix (matrigel: DMEM = 1: 2) was seeded at the bottom of Transwell chamber 1 h prior to the re-suspension of HSC3 cells to let the gel solidify. The number of migrated and invaded cells were qualified by blinded counting of migrated/invaded cells on the lower surface of the membrane, with five fields per chamber, at 24 h and 48 h, respectively.

### 3D culture invasion model and evaluation of carcinoma cell invasion

3D culture invasion model was constructed and based on a method previously described by Igarashi et al. [21, 22]. Matrices containing fibroblasts were prepared by the addition of 1.4 parts of DF12 and 0.6 parts of FBS to 1 part of collagen type I solution (Thermo Fisher, 3 mg/ml), and neutralized with 1 M NaOH solution. Fibroblasts were then embedded into this solution to the final cell density of 5*10^5/ml, and seeded on top with HSC3 at the density of 4.5*10^5/cm^2^ in triplicates. After 5 days of cultivation, 3D constructs were harvested, formalin-fixed, and paraffin-embedded, and sent for slide generation and HE staining.

### Tumor model

To investigate the role of EREG in fibroblasts in vivo, stable EREG over-expression in NFs and EREG knockdown in CAFs were realized by lentivirus vector as previously decribed [17, 23]. Then 10^6^ conditional NFs/CAFs were subcutaneously co-injected with 10^6^ HSC3 in rear flank of four-week old male BALB/c-nu/nu T cells-deficient mice (Cavens, Changzhou, China). Tumor volumes (mm^3^) were measured at various time points until termination of the experiments. Tumors were collected for further experiments. Sixteen mice were used to construct xenografts with HSC3 and fibroblasts (four in each group). At the end of the study, mice were sacrificed and xenografted tumors were harvested. The experiments were executed in compliance with institutional guidelines and regulations.

### Statistic analysis

All images represent at least three independent experiments. The results are presented as the mean ± SEM of at least three independent experiments. Statistical analysis was performed with SPSS® v 19 (SPSS, Chicago, IL, USA). Survival of patients was plotted using the Kaplan–Meier method and analyzed using the log-rank test, with patients censored at last follow-up. Kruskal–Wallis and Mann– Whitney U- and *t*-tests were used to compare groups, as appropriate. *P <* 0.05 was considered statistically significant for all tests (*p < 0.05, **p < 0.01, **p < 0.001, ***p < 0.0001).

## Results

### EREG expression is higher in CAFs than in matched NFs

**Fig. 1:**
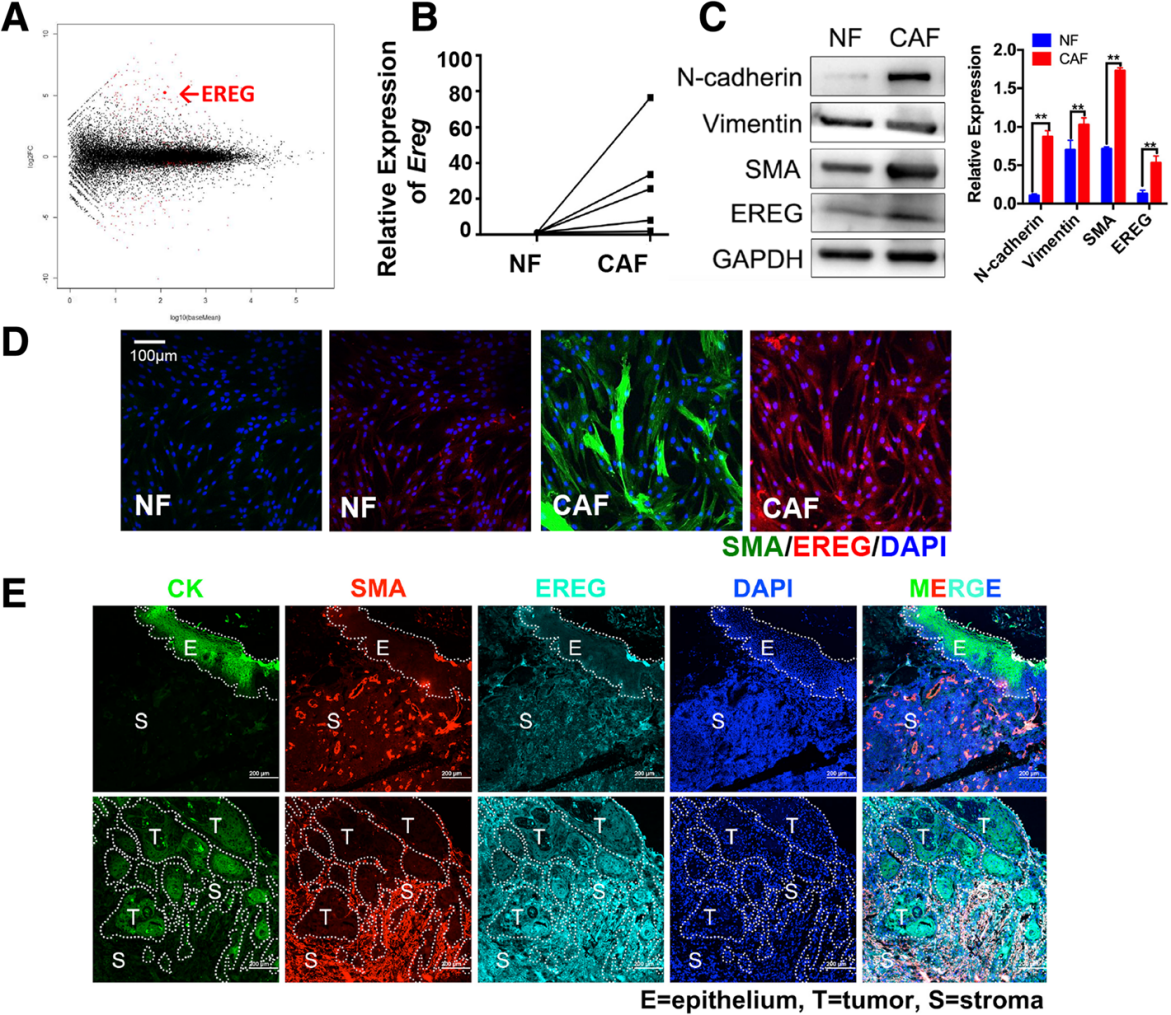
EREG is highly expressed in CAFs. **a**, Gene expression differences between CAFs and NFs (vertical axis) and the average expression of genes in CAFs versus that in NFs (horizontal axis) are presented as a Bland-Altman plot. Data are from our RNA-seq analysis. **b**, Relative *Ereg* mRNA expression was tested in paired NFs/CAFs through quantitative RT-PCR, showing higher expression in CAFs than in NFs. **c**, Representative images and quantitative analysis of western blotting showing that EREG, N-cadherin, vimentin, and SMA were highly expressed in primary cultured CAFs. GAPDH was used as a loading control. **d**, Immunofluorescence staining showing the subcellular location and the expression of SMA and EREG in NFs and CAFs. Magnification: 200×. **e**, Immunofluorescence staining showing that EREG expression was significantly higher in tumor stroma than in the stroma of normal epithelium. Magnification: 200×. **: *p* < 0.01

As an abnormal activated form of NFs, CAFs differ from NFs with distinct phenotypes and functions. Exploration of the regulatory molecules that drive the transition from NFs to CAFs is urgently needed. Therefore, we carried out genomic analysis to identify differentially expressed genes between matching NFs and CAFs obtained from 5 OSCC patients as previously reported [17]. A series of differentially expressed genes were identified, among which *Ereg* was one of the genes with the most significant differential expression (Fig. 1a, Additional file 1: Table S1. The data are the mean value of the 5 matched samples).

To validate the results of the genomic studies, we then examined *Ereg* expression at the gene level in another 5 matching NFs and CAFs by quantitative PCR and found that *Ereg* was significantly higher in CAFs than the matching NFs (Fig. 1b). Furthermore, immunoblotting assays and immunofluorescence results also showed that EREG was significantly higher in CAFs than in matched NFs at the protein level, indicating that EREG may be involved in the transition from NFs to CAFs (Fig. 1c). Furthermore, the EREG expression profiles in normal mucosa tissue and tumor tissue were assessed by immunofluorescence staining in frozen sections. The results demonstrated that EREG expression was also significantly higher in fibroblasts from the cancer stroma (CAFs) than in fibroblasts from the stroma around normal mucosa (NFs; Fig. 1e). These results all proved that EREG expression was much higher in CAFs than in matched NFs.

### High EREG expression in fibroblasts is related to poor prognosis in OSCC patients

**Fig. 2:**
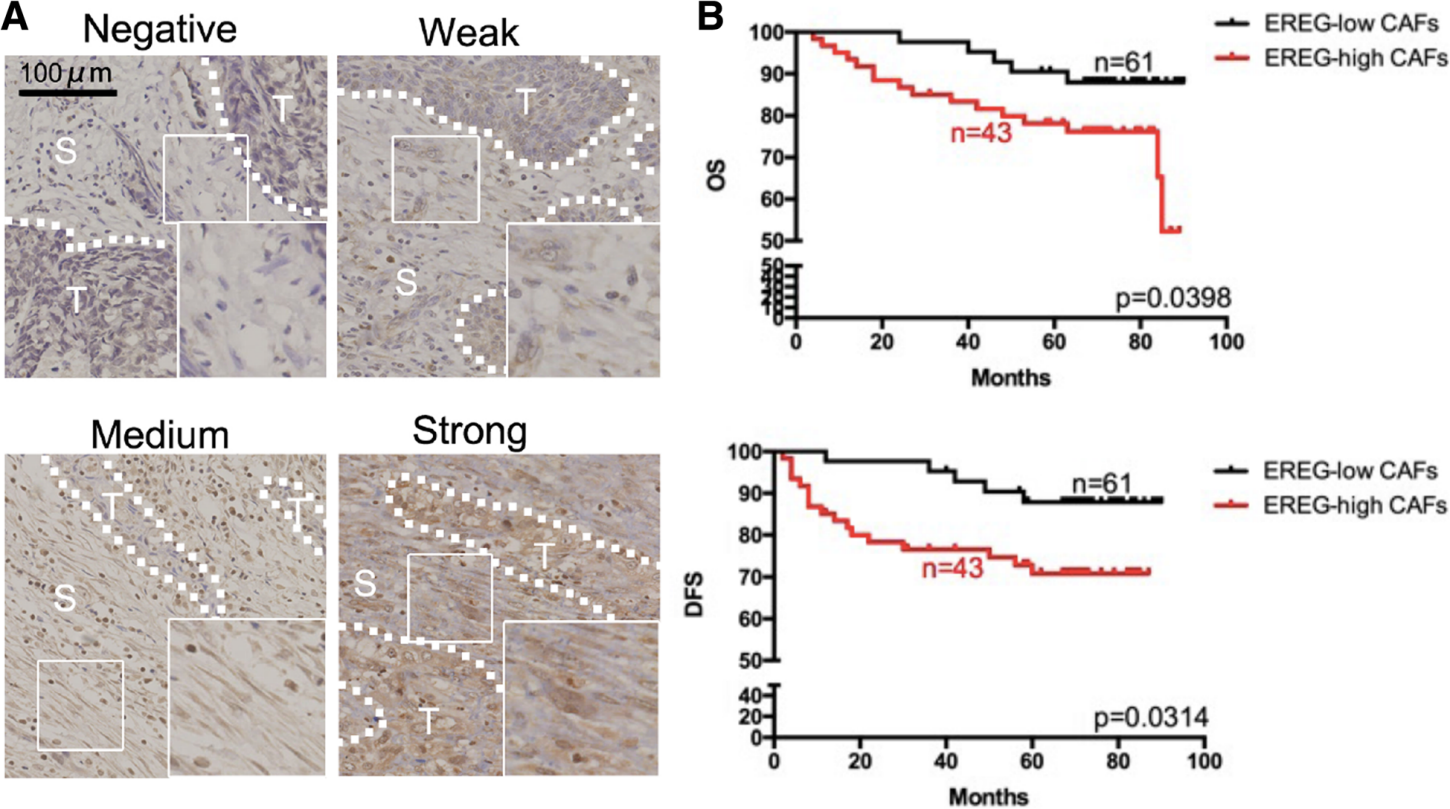
High EREG expression in CAFs of OSCC is related to poor overall survival (OS) and poor disease-free survival (DFS). **a**, Representative IHC images of EREG expression in CAFs from clinical samples. **b**, Survival analysis using Kaplan–Meier curves to compare patients with low or high EREG expression in CAFs from OSCC patients. The results revealed that high EREG expression in CAFs was closely related to inferior overall survival (OS, *p* = 0.0398), and disease-free survival (DFS, *p* = 0.0314)

It is well recognized that CAFs can promote the progression of tumors. However, the clinical outcomes of EREG expression in fibroblasts remain unclear. Considering the high level of EREG in CAFs, we first analyzed the correlation between EREG expression and the clinicopathological characteristics of patients with OSCC (*n* = 104). Chi-square analysis revealed that high EREG expression in CAFs was closely related to higher T staging (*p* = 0.0041), deeper depth of invasion (DOI, *p* < 0.0001) and inferior worst pattern of invasion (WPOI, *p* < 0.0001) (Table 1). Then, the prognostic value of EREG expression in fibroblasts was assessed. Kaplan–Meier analysis showed that high EREG expression in CAFs was significantly correlated with inferior overall survival (OS), and disease-free survival (DFS) (Fig. 2b, *p* = 0.0398, and *p* = 0.0314). The results demonstrated that high EREG expression in fibroblasts correlated with poor prognosis in OSCC patients.

### EREG promotes the transition from NFs to CAFs

**Fig. 3:**
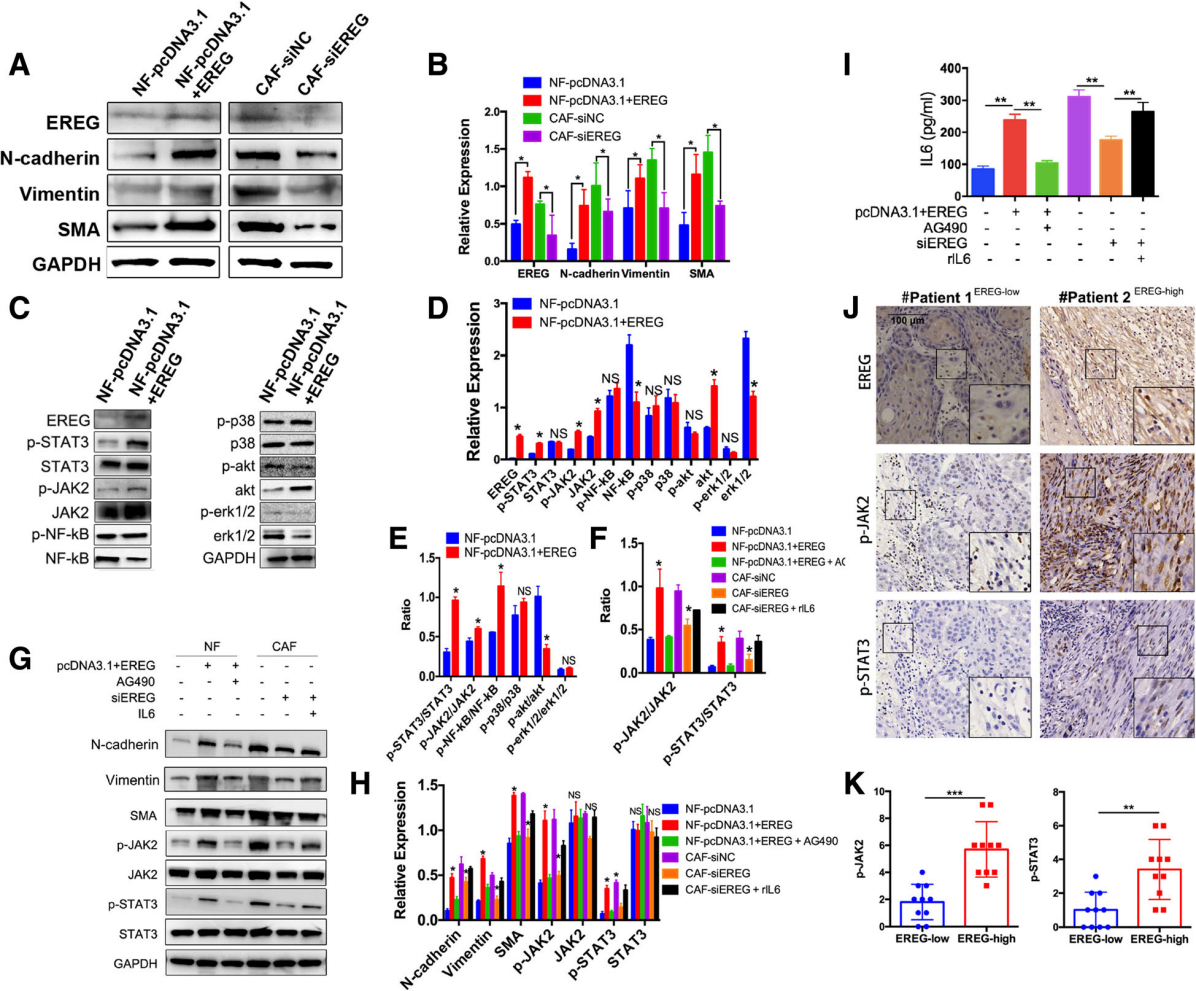
High EREG expression promotes NF-CAF transition through the JAK2-STAT3 pathway. **a**&**b**, Representative images and quantitative analysis of western blotting showing that EREG overexpression in NFs and EREG interference in CAFs were successful. Moreover, the expression of CAF markers, including N-cadherin, vimentin and SMA, was upregulated after EREG overexpression and downregulated after EREG interference. GAPDH was used as a loading control. **c** & **d**& **e**, Both phosphorylated forms of JAK2 and STAT3 and the p-JAK2/JAK2 and p-STAT3/STAT3 ratios were significantly augmented after EREG overexpression. The change in expression of major participants of other pathways after EREG overexpression was not as significant. GAPDH was used as a loading control. **f** & **g**& **h**, Representative images and quantification of western blotting showing that expression of CAF markers, including N-cadherin, vimentin and SMA, was upregulated after EREG overexpression but was reduced after treatment with the JAK2 inhibitor AG490, indicating that AG490 antagonizes EREG-mediated NF activation. CAF markers, as well as (p-)JAK2 and (p-)STAT3 expression and p-JAK2/JAK2 and p-STAT3/STAT3 ratios decreased after EREG interference in CAFs but were restored by IL-6 treatment. GAPDH was used as a loading control. **i**, ELISA showing the IL-6 level in the CM of NFs/CAFs after the indicated treatment. J&K, Representative images and quantification of immunohistochemical staining in clinical samples revealed that high EREG expression was correlated with high phospho-JAK2 and phospho-STAT3 expression, while low EREG expression was correlated with low phospho-JAK2 and phospho-STAT3 expression. Magnification: 200×. *: *p* < 0.05, **: *p* < 0.01, ***: *p* < 0.001

In addition, we ascertained the role of EREG during the NF-CAF transition. First, we modified EREG expression in NFs and CAFs separately and evaluated changes in the cell phenotypes. High N-cadherin, vimentin, and SMA expression was used to mark the CAF phenotype. Three siRNAs (siEREG-1, siEREG-2, and siEREG-3) were designed, and their interference rates were 64.9, 58.3, and 48.9%, respectively. Then, siRNA-1, which had the highest interference rate, was chosen for subsequent experiments (hereafter referred to as siEREG; Additional file 2: Figure S1B). Plasmid overexpression of EREG in NFs was also successful (Additional file 2: Figure S1A). In addition to the confirmation of EREG expression at the gene level, we also confirmed the overexpression and interference of EREG by ELISA at the protein level (Additional file 2: Figure S1C). Overexpression of EREG in NFs significantly augmented the expression of N-cadherin, vimentin, and SMA, while siRNA modulation of EREG levels in CAFs resulted in reduced N-cadherin, vimentin, and SMA expression (Fig. 3a&b), indicating that EREG was involved in the transition from NFs to CAFs. However, the specific mechanism underlying EREG participation in the NF-CAF transition remains to be further elucidated.

### EREG regulates the NF-CAF transition via the JAK2-STAT3 pathway

To ascertain the pathway of the EREG-regulated NF-CAF transition, major pathways, including the JAK2-STAT3 pathway, NF-kB pathway, p38-MAPK pathway, PI3K/AKT pathway and erk1/2 pathway, were tested in unmodified NFs and EREG-overexpressing NFs. The results showed that both the levels of phospho-JAK2 and phospho-STAT3 and the ratios of phospho-JAK2/JAK2 and phospho-STAT3/STAT3 were significantly increased by the overexpression of EREG in NFs, indicating that the JAK2-STAT3 pathway was the most activated pathway after EREG overexpression (Fig. 3c-e). Although a close relationship between the erk1/2 pathway and EREG has been reported [13], our results demonstrated that erk1/2 did not play a significant role in the EREG-mediated NF-CAF transition (Fig. 3c-e).

To further confirm the role of the JAK2-STAT3 pathway in the EREG-mediated NF-CAF transition, we treated EREG-overexpressing NFs with AG490, an inhibitor of the JAK2-STAT3 pathway, and found that AG490 could reverse EREG-mediated CAF marker augmentation (Fig. 3f-h). Moreover, AG490 reduced EREG-mediated CAF activation (Additional file 3: Figure S2A). IL-6 is an important downstream factor of the JAK2-STAT3 pathway [24]. To further confirm the role of the JAK2-STAT3 pathway, we added recombinant human IL-6 (rIL-6) into the culture medium to construct a rescue assay. The results showed that while siEREG significantly lowered the expression of N-cadherin, vimentin and SMA, rIL-6 upregulated their expression (Fig. 3f-h). IL-6 levels were also assessed by ELISA. The results demonstrated that the IL-6 level was increased after EREG overexpression and was downregulated after EREG interference (Fig. 3i). In clinical OSCC samples, we also found that high EREG expression in CAFs was correlated with higher phospho-JAK2 and phospho-STAT3 expression (Fig. 3j-k). These results all demonstrate that EREG activates NFs in a JAK2-STAT3-dependent way. In addition to the change in protein markers, in OSCC, it has been reported that CAFs promote tumor migration [25] and invasion [7, 26] and are related to poor prognosis [27]. However, the effects of the EREG-mediated NF-CAF transition during OSCC progression are still unknown.

### CAFs-derived EREG promotes the invasion and migration of OSCC cells

**Fig. 4:**
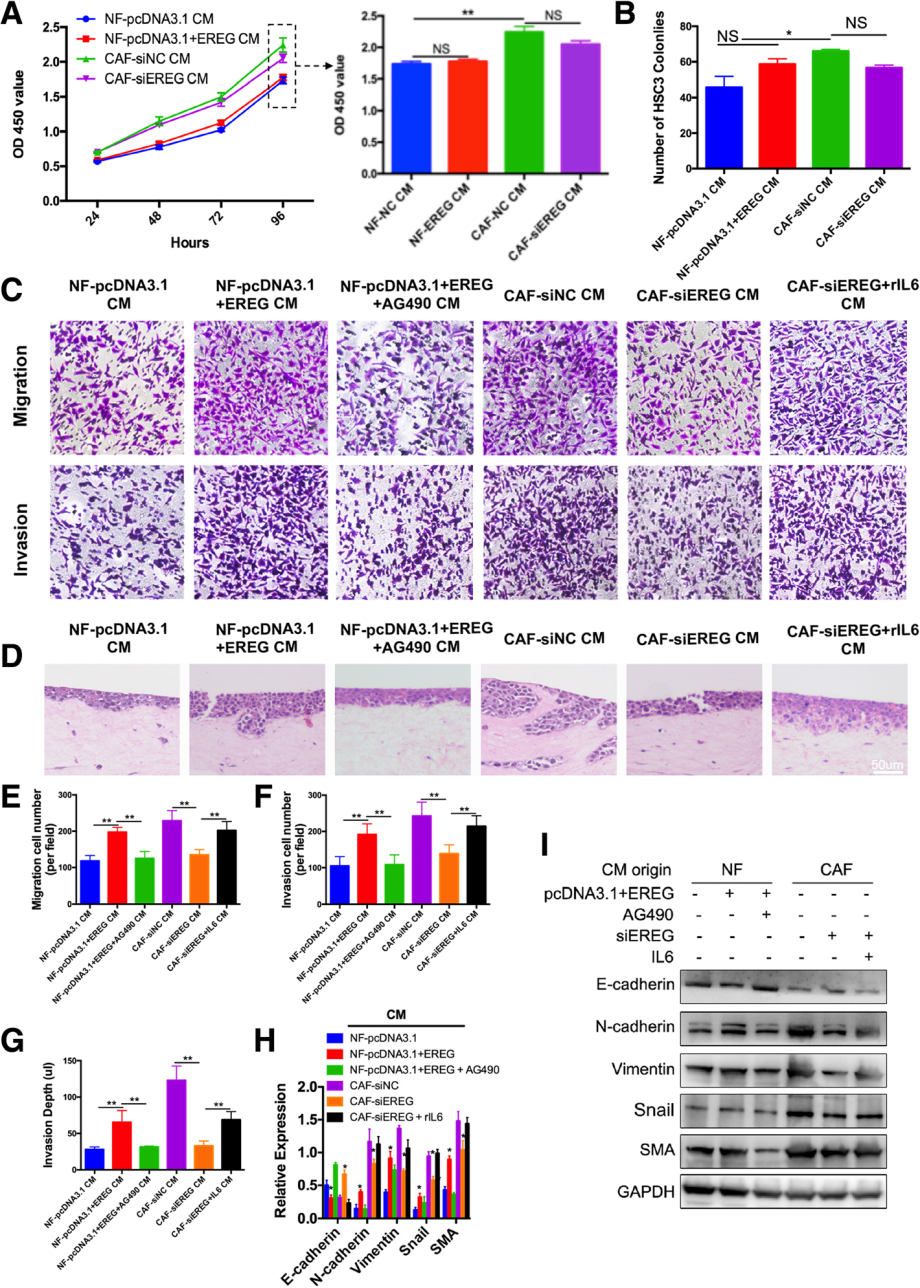
NFs/CAFs with modulated EREG expression regulate the migration and invasion of oral squamous cell carcinoma cell lines. **a**, CCK8 assay showing that CAF CM significantly promoted HSC3 cell viability compared with NF CM. However, alteration of EREG expression in the same type of fibroblasts did not significantly affect their influence on HSC3 cell viability. **b**, Quantification of colony formation revealed that CAF-siNC CM significantly promoted HSC3 proliferation compared with NF-pcDNA3.1 CM. However, alteration of EREG expression in the same type of fibroblasts did not significantly affect their influence on HSC3 proliferation. **c**, Representative images and transwell migration and invasion assays with the HSC3 cell line. EREG upregulation induced migration/invasion-promoting CAF-like functions in NFs, but this effect was attenuated by AG490 treatment. On the other hand, EREG downregulation in CAFs interfered with their migration/invasion-promoting ability but was restored by rIL-6 treatment. **d**, Representative HE staining images of a 3D invasion model using tissue engineering. The results show that CAFs significantly promoted HSC3 invasion compared with NFs, but their invasion-supportive ability was attenuated after EREG knockdown. Treatment with rIL-6 restored the invasion-promoting abilities of EREG-low CAFs. On the other hand, EREG overexpression in NFs led to an increased invasion-promoting ability, but this effect was attenuated by AG490 treatment. **e**-**g**, Quantification of transwell assays and 3D invasion assays. H&I, Representative images and quantification of EMT marker (E-cadherin, N-cadherin, vimentin, SMA, and Snail) expression in HSC3 cells after the indicated treatment. GAPDH was used as a loading control. *: *p* < 0.05, **: *p* < 0.01

Then we investigated whether EREG expression in fibroblasts affected their tumor supportive functions. *Ereg*-expression level was assessed in 4 human OSCC cell lines and one normal epithelium cell line HACAT by qPCR. All 4 OSCC cell lines showed higher *Ereg* expression level compared with HACAT, and HSC3 was chosen for further experiments as it expressed the lowest level of *Ereg* among OSCC cell lines (Additional file 2: Figure S1D). Then, CM obtained from NFs/CAFs were added in to HSC3, and we found that compared with CM obtained from NFs, CM obtained from CAFs significantly improved the cell viabilities and promoted proliferation of HSC3 (Fig. 4a&b). However, modulation of EREG-expression in NFs/CAFs (transfected with plasmid or siRNA as indicated) did not significantly affect their supporting role of HSC3 viabilities and proliferation (Fig. 4a&b).

In our retrospective study of immunohistochemistry EREG staining in CAFs of OSCC, we already found that high EREG expression in CAFs of OSCC was correlated to a deeper invasion depth and inferior WPOI (Table 1). To confirm these results in vitro and to study the EREG-mediated NF activation in terms of their invasion-supportive abilities, in transwell migration assays, we found that CAFs with EREG interfered had an attenuated effect in inducing HSC3 migration, while up-regulation of EREG in NFs augmented their migration inducing ability (Fig. 4c&e). Moreover, the inhibition of JAK2-STAT3 pathway by AG490 could attenuate the migration-supportive role of EREG-high NFs, while rIL6 could rescue the migration-supportive role of EREG-low CAFs. The invasion abilities of HSC3 were assessed by transwell invasion assays. EREG-overexpressed NF increased HSC3 cell invasion more than two-folds than NF-control, while AG490 inhibited this invasion-supportive ability. Invasion-supportive ability was attenuated in CAF-siEREG, but could be rescued by rIL6 (Fig. 4c&f). Again, AG490 treatment attenuated the EREG-mediated migration and invasion-supportive abilities of CAFs (Additional file 3: Figure S2B).

To further confirm the invasion-inducing ability of EREG, we constructed a 3D invasion model with conditional NFs/CAFs, and found that augmented EREG expression in NFs led to deeper invasion. CAFs supported HSC3 invasion most significantly, with deeper invasion and more diffused spreading. This ability was attenuated after EREG interference, but could be restored by rIL6 (Fig. 4d&g). Meanwhile, overexpression of EREG in NFs significantly increased their invasion-supportive ability, but could be interfered after inhibition of JAK2-STAT3 pathway through AG490 (Fig. 4d&g).

To further reveal the mechanism underlying the EREG-mediated invasion-supportive role of fibroblasts, we analyzed the epithelial-mesenchymal transition (EMT) markers of HSC3 cells after treatment with CM from conditional NFs/CAFs. The results showed that compared with NFs, CAFs could significantly promote EMT in HSC3 cells in an EREG- and JAK2-STAT3-dependent way (Fig. 4h&i).

Together, these results indicate that the NF-CAF transition, which is induced by EREG, affects the migration and invasion of OSCC cells through EMT in a JAK2-STAT3-dependent way.

### EREG expression in fibroblasts promotes OSCC progression with stronger invasive character in vivo

**Fig. 5:**
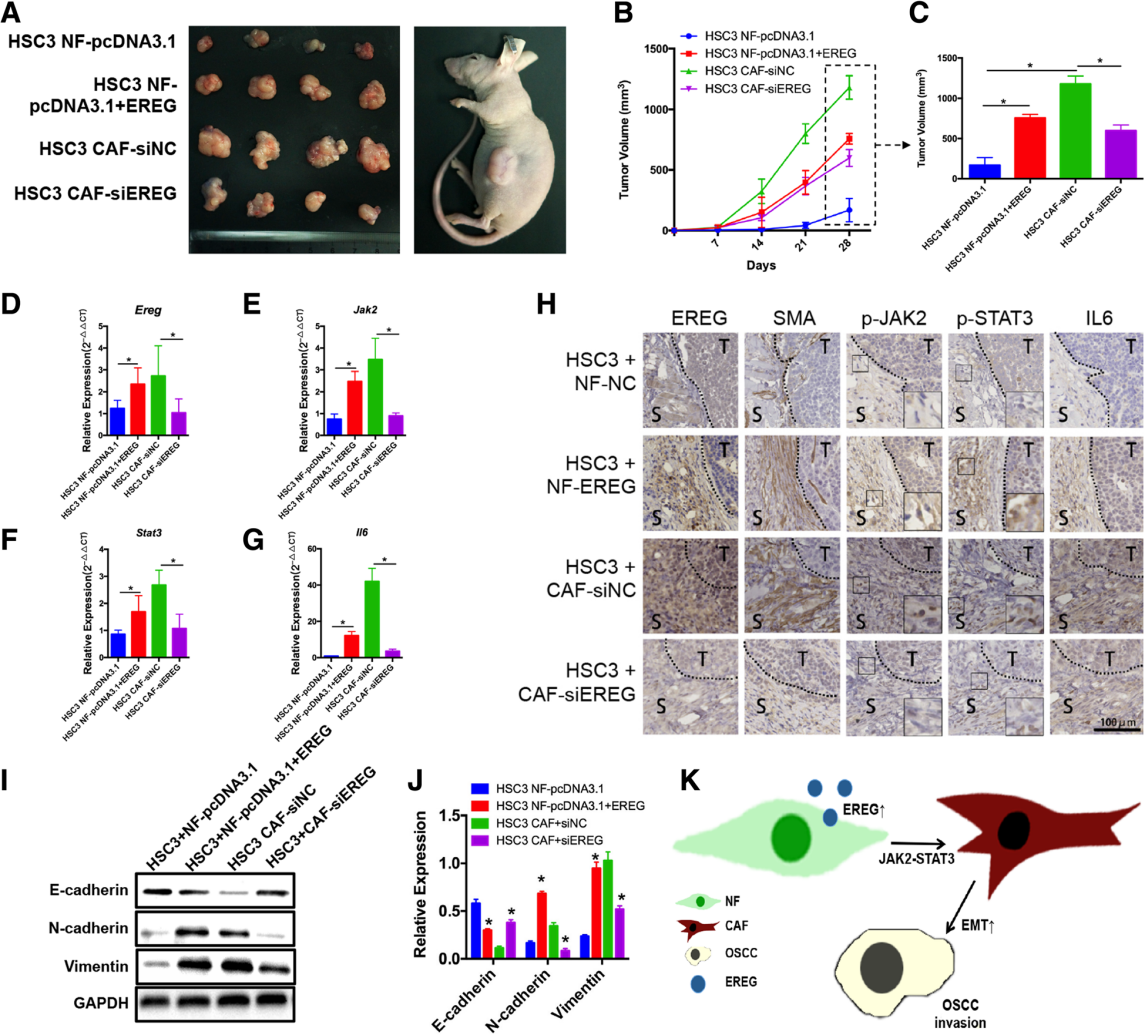
High EREG expression in fibroblasts promotes tumor growth in vivo. **a**, Photographs of tumor formation in nude mice and tumor xenografts 4 weeks after inoculation. **b** & **c**, Tumor growth curves measured after injection of HSC3 cells or conditioned fibroblasts as indicated. The tumor volume was calculated every 7 days until termination. **d**-**g**, RT-PCR analysis of *Ereg, Jak2, Stat3* and *Il6* expression in tissues of resected tumors, revealing successful overexpression/interference of *Ereg* and associated upregulation or downregulation of *Jak2, Stat3* and *Il6*. **h**, Immunohistochemical staining showed that tumors that developed from EREG-overexpressing NFs had a higher level of EREG, phospho-JAK2, phospho-STAT3, and IL-6 protein expression than tumors that developed from control NFs. Tumors that developed from siEREG-transfected CAFs showed a lower level of EREG, phospho-JAK2, phospho-STAT3, and IL-6 protein expression than tumors developed from siNC-transfected CAFs. S: stroma, T: tumor. Magnification: 200×. **i** & **j**, Western blotting showed that CAFs significantly promote EMT in vivo compared with NFs, with decreased E-cadherin expression and increased N-cadherin and vimentin expression. This EMT-promoting ability was attenuated after EREG knockdown. On the other hand, EREG overexpression in NFs gave NFs a CAF-like EMT-promoting ability. **k**, Increased EREG expression in NFs led to acquisition of the CAF phenotype in a JAK2-STAT3-dependent way and supported OSCC invasion through the promotion of EMT in tumor cells

To further examine the tumorigenic role of EREG in vivo, we established a xenograft model in which 1*10^6^ HSC3 cells were subcutaneously coinjected with 10^6^ conditioned fibroblasts into the flank of nude mice, which were allowed to develop measurable tumors. One of the most important functional characteristics of CAFs is their tumor-supportive nature in vivo. Indeed, HSC3 cells coinjected with CAFs demonstrated rapid in vivo tumor growth, but this tumor-supportive effect could be attenuated by EREG knockdown (Fig. 5a-c). On the other hand, NFs demonstrated an in vivo tumor-supportive role after EREG overexpression (Fig. 5a-c). EREG knockdown and EREG overexpression were confirmed by qPCR and IHC (Fig. 5d & h). The EREG-mediated NF-CAF transition was confirmed in vivo, with IHC showing a positive correlation between EREG expression and SMA expression in fibroblasts (Fig. 5h). Moreover, EREG modulated the NF-CAF transition through JAK2-STAT3 pathway activation (Fig. 5e-h). In addition, WB confirmed that EREG regulated the EMT-promoting abilities of fibroblasts in vivo (Fig. 5i&j). Thus, these data provide novel insights into the involvement of EREG in the EREG-JAK2-STAT3-IL-6 axis in fibroblasts, which supports OSCC invasion through induction of EMT in the tumor microenvironment.

## Discussion

Available evidence suggests that CAFs, as an irreversible active form of NFs, promote the progression of OSCC [6]. However, the molecular mechanisms underlying the NF-CAF transition remain largely unclear.

The activation of NFs occurs through a multistep process. During wound healing, spindle-shaped quiescent resting fibroblasts (NFs) are reversibly activated to facilitate repair and regeneration. Normal activated fibroblasts (NAFs) gain smooth muscle actin (SMA) and vimentin and became stellate in shape. Activated fibroblasts can be further activated until there is “no way back” and they finally transit to CAFs. Such CAFs gain enhanced proliferative properties and are a functionally diverse population, adding to the dynamic complexity of the tumor microenvironment milieu [28].

To identify key participants in NF-CAF reprogramming, 5 pairs of matching NFs and CAFs were sent for genomic analysis. We reviewed genes with the most significant changes and identified epiregulin (EREG) as a strong candidate. From its initial discovery, EREG has been closely linked with fibroblasts [29]. It has been reported as the most significantly altered gene between NFs and CAFs in other studies [6, 13]. It is overexpressed not only in CAFs but also in the stroma during wound healing [13, 15], indicating that it could take part in NF activation from the very start, activating even the most quiescent NFs.

In a cohort consisting of 104 OSCC patients, we found that high levels of EREG expression in fibroblasts were significantly correlated with poorer OS and DFS, deeper DOI and higher levels of WPOI in OSCC patients. DOI and WPOI are prominent indicators for tumor invasion and are closely related to OSCC prognosis [30, 31]. These results indicate that EREG secreted by CAFs is related to inferior OSCC prognosis and promotes OSCC invasion.

Next, we identified the causal relationship between EREG expression in fibroblasts and their CAF-like phenotype. Overexpression of EREG led to the activation of NFs, resulting in augmented expression of CAF markers (SMA, N-cadherin, and vimentin) and upregulated cell viability. Moreover, CAFs, a naturally irreversible activated form of NFs, were deprived of their CAF phenotype by EREG knockdown.

Through years of study, the tumor-promoting functions of the JAK2-STAT3 pathway have been well recognized in various malignancies [32]. It has been reported that JAK-STAT can promote the proliferation [33] and activation of fibroblasts [34]. IL-1 induces the JAK-STAT pathway to shape CAF heterogeneity in pancreatic ductal adenocarcinoma [35]. CAFs promote bladder cancer EMT via paracrine IL-6 [36]. However, further evidence is needed regarding JAK-STAT-mediated fibroblast activation in oral cancer. Our study revealed that, in the abovementioned EREG-mediated NF activation, the JAK2-STAT3 pathway is the most significantly activated pathway. EREG could activate fibroblasts in a JAK2-STAT3-dependent way.

In addition to protein marker alteration, a widely recognized functional characteristic of CAFs is their supportive role in tumor progression. Existing evidence supports the idea that EREG is involved in carcinogenesis [12, 13] and metastasis [37] and correlates with poor prognosis [38, 39]. Fibroblast-derived EREG promotes epithelial cell proliferation in cholesteatoma [11]. In our study, even though the EREG level was positively correlated with fibroblast proliferation ability, colony formation and CCK8 assays revealed that modulating EREG in NFs/CAFs did not significantly alter their effects on oral cancer cell proliferation. On the other hand, wound-healing assays, transwell assays and 3D invasion models demonstrated that altered EREG expression in fibroblasts significantly influences their supportive role in cancer cell migration and invasion, with more significant effects on invasion. High EREG levels in fibroblasts could promote their invasion-supportive abilities by encouraging OSCC EMT in a JAK2-STAT3 pathway-dependent way.

Last, we confirmed our results in vivo*.* In a xenograft tumor model, EREG expression in fibroblasts had a tumor-supportive function in an EREG-related pattern. Phospho-JAK2 and phospho-STAT3 levels were related to EREG expression.

In recent years, CAFs have become a novel target in cancer treatment [40–42]. The identification of EREG as a pivotal participant in the NF-CAF transition highlights critical pathways that could be targeted for novel therapeutic interventions.

## Conclusions

In this study, we showed that EREG is an important protein that could reprogram NFs into CAFs via the JAK2-STAT3 pathway. The EREG expression level was related to the invasion-supportive role of fibroblasts and predicted a poor clinical outcome of OSCC patients. Thus, EREG overexpression in fibroblasts is implicated in the NF-CAF transition and in OSCC progression (Fig. 5k).

## Additional files

Additional file 1: **Table S1.** The levels and fold-changes of differentially expressed mRNAs. **Table S2.** Primer sequences. (DOC 133 kb)

Additional file 2: **Figure S1.** A, Relative *Ereg* mRNA levels in NFs after transfection with the control plasmid pcDNA3.1 or the EREG-overexpressing plasmid pcDNA3.1-EREG, showing successful EREG overexpression in NFs. B, Relative EREG mRNA levels in NFs after transfection with control siRNA (siNC) or EREG interference siRNA. EREG siRNA-1 (hereby referred to as siEREG) was chosen for further experiments. C, ELISA showing increased EREG expression of NFs after EREG overexpression and decreased EREG expression of CAFs after EREG interference. D, *Ereg* mRNA expression was analyzed in 1 normal epithelial cell line (HACAT) and 4 different oral squamous cell carcinoma cell lines (OSCC3, SCC4, HSC3, and Cal27) by quantitative RT-PCR. HSC3 cells, having the lowest *Ereg* mRNA level, were chosen for further research. (TIF 3286 kb)

Additional file 3: **Figure S2.** AG490 reduces EREG-mediated CAF activation. AG490 was added to the CM of CAFs transfected with pcDNA3.1-EREG, and treatment was carried out for 48 h. Then, CAFs were sent for protein extraction for WB analysis (A) or seeded for transwell assays (B). The results revealed that AG490 reduced EREG-mediated CAF activation (A) and pro-migration and pro-invasion abilities (B). (TIF 1444 kb)

## Abbreviations

AJCC: American Joint Committee on Cancer
CAF: cancer-associated fibroblasts
CM: conditioned medium
DOI: depth of invasion
EGF: epidermal growth factor
EGFR: epidermal growth factor receptor
EMT: epithelial-mesenchymal transition
EREG: epiregulin
NF: normal fibroblast
OSCC: oral squamous cell carcinoma
TNM: tumor–node–metastasis TNM
WPOI: worst pattern of invasion
