# Supplementary material for: Epiregulin reprograms cancer-associated fibroblasts and facilitates oral squamous cell carcinoma invasion via JAK2-STAT3 pathway
Source: J Exp Clin Cancer Res. 2019 Jun 24;38:274. doi: 10.1186/s13046-019-1277-x (PMC6591968; doi:10.1186/s13046-019-1277-x)
Supplement: Supplementary file 1 — Table S1. The levels and fold-changes of differentially expressed mRNAs. Table S2. Primer sequences. (DOC 133 kb) [file 13046_2019_1277_MOESM1_ESM.doc]

**Table S1. The levels and fold-changes of differentially expressed mRNAs.**

| AccID | CAF | NF | Log2FC | FDR | Style |
| --- | --- | --- | --- | --- | --- |
| MYH7 | 123.7187219 | 0.201559099 | 9.261645205 | 4.96597E-09 | up |
| MYOG | 432.7548599 | 1.352910206 | 8.321340121 | 2.08772E-10 | up |
| CHRND | 51.28956429 | 0.201559099 | 7.991318591 | 4.77002E-05 | up |
| MYOD1 | 362.8433752 | 1.751661091 | 7.694479448 | 3.16873E-09 | up |
| MYH8 | 62.84114816 | 0.304522726 | 7.689015832 | 3.33183E-05 | up |
| CHRNG | 157.1203739 | 0.806236397 | 7.606451638 | 4.11323E-06 | up |
| MYL2 | 37.29395918 | 0.201559099 | 7.531595239 | 4.60369E-05 | up |
| BPIFB4 | 51.02524733 | 0.403118199 | 6.983864545 | 0.000175681 | up |
| MYH2 | 64.96328823 | 0.546673809 | 6.892800591 | 7.94709E-07 | up |
| TNNI1 | 562.2125708 | 4.931705133 | 6.832885358 | 1.32878E-08 | up |
| SBK2 | 21.00696808 | 0.201559099 | 6.703521229 | 0.002939389 | up |
| ANGPTL7 | 20.45279113 | 0.201559099 | 6.664951012 | 0.000497307 | up |
| SLC47A2 | 27.82164356 | 0.275520562 | 6.657903851 | 0.004524811 | up |
| UNC5D | 20.09158335 | 0.201559099 | 6.639244537 | 0.014712852 | up |
| SLC7A4 | 167.5830316 | 1.722659766 | 6.604094478 | 7.73315E-08 | up |
| TRIM72 | 19.17297808 | 0.201559099 | 6.571727719 | 0.000977774 | up |
| KLHL41 | 571.0982449 | 6.16855061 | 6.532663594 | 5.45001E-08 | up |
| EPYC | 18.5998245 | 0.201559099 | 6.527942284 | 0.009766735 | up |
| CHRDL2 | 112.4462784 | 1.386280524 | 6.341872876 | 3.30261E-05 | up |
| MYLPF | 215.9306783 | 2.705820412 | 6.318358328 | 3.76376E-07 | up |
| SHD | 91.44659985 | 1.209354596 | 6.240620303 | 4.42416E-05 | up |
| PI16 | 939.6641951 | 13.30856242 | 6.141718631 | 8.5854E-05 | up |
| TNNT3 | 56.47506776 | 0.806236397 | 6.130267372 | 1.40317E-05 | up |
| MYL1 | 12.77509763 | 0.201559099 | 5.985987592 | 0.0275035 | up |
| MYH1 | 62.05907541 | 1.019386156 | 5.927869633 | 8.47826E-05 | up |
| MYCL | 88.05499003 | 1.487731188 | 5.887218983 | 2.66078E-06 | up |
| SFRP5 | 51.45065105 | 0.88019786 | 5.869217659 | 0.000996784 | up |
| CLDN5 | 35.11069397 | 0.604677298 | 5.859601286 | 0.002374648 | up |
| VGLL2 | 11.50605519 | 0.201559099 | 5.83504657 | 0.012638499 | up |
| PCSK1 | 34.12045692 | 0.604677298 | 5.818327741 | 0.002763856 | up |
| TTN | 958.6959959 | 17.00492581 | 5.81704879 | 0.000105404 | up |
| LMOD3 | 33.37514556 | 0.604677298 | 5.786464903 | 0.000459752 | up |
| CDH15 | 282.0179995 | 5.317354075 | 5.728934897 | 1.05263E-06 | up |
| HFE2 | 26.09707702 | 0.506081825 | 5.688373754 | 0.000687868 | up |
| TMEM8C | 112.7286441 | 2.217150093 | 5.668003899 | 5.78948E-05 | up |
| MYBPH | 297.8910433 | 5.948298048 | 5.646163999 | 7.81636E-07 | up |
| CHI3L1 | 12142.72214 | 256.5850482 | 5.564510966 | 7.29282E-06 | up |
| EREG | 179.1068906 | 4.03555014 | 5.471911672 | 0.000184511 | up |
| KCNB1 | 93.57758615 | 2.179471891 | 5.424112512 | 0.000907901 | up |
| APELA | 20.02171787 | 0.542306495 | 5.206313505 | 0.015618238 | up |
| SLC8A3 | 21.95205781 | 0.604677298 | 5.18204696 | 0.008671793 | up |
| RAPSN | 241.9180888 | 6.66740909 | 5.181248588 | 1.57798E-05 | up |
| TSHR | 9.830639966 | 0.275520562 | 5.157053444 | 0.026810705 | up |
| DES | 3719.645379 | 109.2676897 | 5.089226319 | 7.6066E-06 | up |
| ACTN2 | 168.3785597 | 4.994078456 | 5.07534615 | 0.000212984 | up |
| RYR1 | 135.6777044 | 4.224065048 | 5.005407804 | 0.000323467 | up |
| RGS5 | 189.0236049 | 6204.851491 | -5.036758369 | 2.62493E-05 | down |
| LGR6 | 3.929494573 | 147.6167988 | -5.23136934 | 9.60234E-05 | down |
| TCN1 | 1.124123891 | 46.43416014 | -5.36831359 | 0.000388178 | down |
| KLB | 0.205991289 | 8.667041271 | -5.39488434 | 0.029371281 | down |
| TCHH | 1.57348447 | 69.89022732 | -5.473055895 | 8.50556E-05 | down |
| GPR110 | 0.749415927 | 33.86984658 | -5.498091002 | 0.000219875 | down |
| CTNNA2 | 0.169953936 | 7.860804874 | -5.531461359 | 0.045121107 | down |
| CA8 | 3.229124784 | 150.0497672 | -5.538154077 | 1.29616E-05 | down |
| ATP2B2 | 0.339907872 | 16.02613247 | -5.559138723 | 0.004126982 | down |
| KRT15 | 16.22823804 | 786.8673817 | -5.59954223 | 6.02593E-05 | down |
| OR51E2 | 0.991606158 | 48.62067252 | -5.615658808 | 0.000789153 | down |
| PSG4 | 118.475253 | 6009.743982 | -5.664645885 | 0.000113486 | down |
| ST8SIA6 | 1.955068165 | 100.3764315 | -5.682057843 | 3.01449E-05 | down |
| GATA3 | 0.392807435 | 20.28817372 | -5.690672958 | 0.000356419 | down |
| HS6ST3 | 0.187353982 | 9.97237639 | -5.734098791 | 0.046893659 | down |
| RANBP3L | 6.244256841 | 365.3367879 | -5.870553344 | 5.19205E-06 | down |
| PRSS35 | 34.90919104 | 2262.142466 | -6.017939055 | 2.93772E-06 | down |
| GLP1R | 0.372238441 | 24.59021012 | -6.045713203 | 0.000474028 | down |
| ST6GALNAC1 | 0.169953936 | 11.45080719 | -6.074161716 | 0.02437665 | down |
| SPHKAP | 0.411444742 | 30.85830289 | -6.228816225 | 0.000604525 | down |
| SYT9 | 0.562061946 | 47.38516927 | -6.397562641 | 0.000142357 | down |
| PAX2 | 0.169953936 | 18.0127213 | -6.727728571 | 0.000856035 | down |
| CRNN | 0.187353982 | 21.87214885 | -6.867184516 | 0.000673848 | down |
| KRT4 | 1.554743776 | 294.4581481 | -7.565241944 | 9.71971E-08 | down |
| KRT13 | 6.744743347 | 1380.792348 | -7.677517113 | 1.54446E-08 | down |
| SLC6A12 | 0.169953936 | 178.3841711 | -10.03562811 | 2.89551E-09 | down |

**Table S2.** Primer sequences

| Primer name | Primer sequences (5’→3’) |
| --- | --- |
| *Gapdh* | Forward: GGAGCGAGATCCCTCCAAAAT |
|  | Reverse: GGCTGTTGTCATACTTCTCATGG |
| *Ereg* | Forward: GTGATTCCATCATGTATCCCAGG |
|  | Reverse: GCCATTCATGTCAGAGCTACACT |
| *Sma* | Forward: CCTGTGTTGTGGTTTACACTGG |
|  | Reverse: GGGGGAATTATCTTTCCTGGTCC |
| *Jak2* | Forward: TCTGGGGAGTATGTTGCAGAA |
|  | Reverser: AGACATGGTTGGGTGGATACC |
| *Stat3* | Forward: ACCAGCAGTATAGCCGCTTC |
| *Il6* | Reverse: GCCACAATCCGGGCAATCT  Forward: ATGGATGCTACCAAACTGGAT  Reverse: TGAAGGACTCTGGCTTTGTCT |
