# Supplementary figures and images for: Epiregulin reprograms cancer-associated fibroblasts and facilitates oral squamous cell carcinoma invasion via JAK2-STAT3 pathway
Source: J Exp Clin Cancer Res. 2019 Jun 24;38:274. doi: 10.1186/s13046-019-1277-x (PMC6591968; doi:10.1186/s13046-019-1277-x)

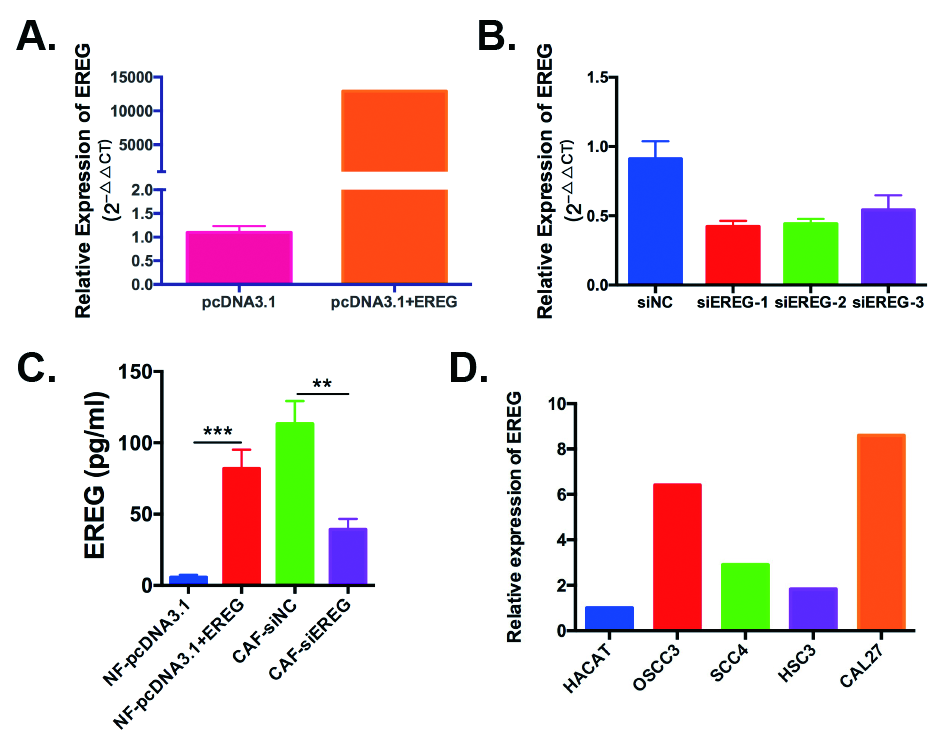

Supplement: Supplementary file 2 — Figure S1. A, Relative Ereg mRNA levels in NFs after transfection with the control plasmid pcDNA3.1 or the EREG-overexpressing plasmid pcDNA3.1-EREG, showing successful EREG overexpression in NFs. B, Relative EREG mRNA levels in NFs after transfection with control siRNA (siNC) or EREG interference siRNA. EREG siRNA-1 (hereby referred to as siEREG) was chosen for further experiments. C, ELISA showing increased EREG expression of NFs after EREG overexpression and decreased EREG expression of CAFs after EREG interference. D, Ereg mRNA expression was analyzed in 1 normal epithelial cell line (HACAT) and 4 different oral squamous cell carcinoma cell lines (OSCC3, SCC4, HSC3, and Cal27) by quantitative RT-PCR. HSC3 cells, having the lowest Ereg mRNA level, were chosen for further research. (TIF 3286 kb) [file 13046_2019_1277_MOESM2_ESM.tif]

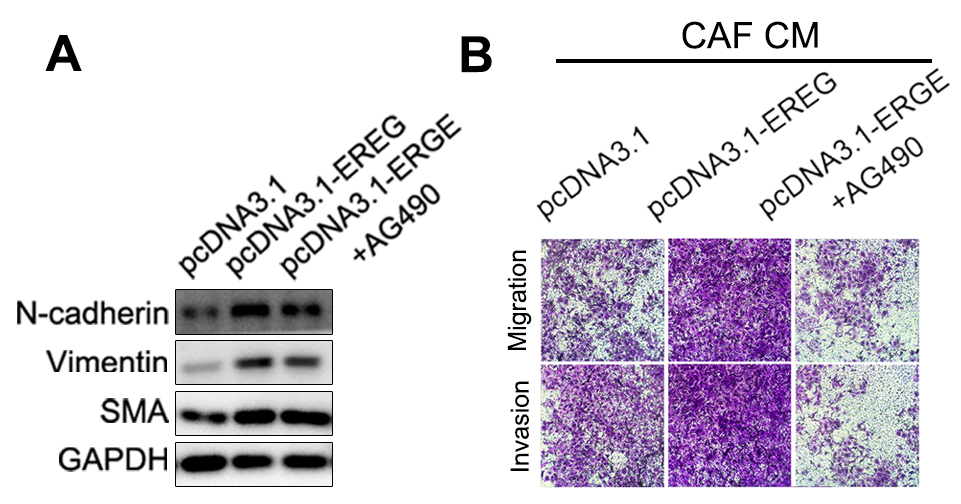

Supplement: Supplementary file 3 — Figure S2. AG490 reduces EREG-mediated CAF activation. AG490 was added to the CM of CAFs transfected with pcDNA3.1-EREG, and treatment was carried out for 48 h. Then, CAFs were sent for protein extraction for WB analysis (A) or seeded for transwell assays (B). The results revealed that AG490 reduced EREG-mediated CAF activation (A) and pro-migration and pro-invasion abilities (B). (TIF 1444 kb) [file 13046_2019_1277_MOESM3_ESM.tif]
